# Asymmetric Localization of CK2α During *Xenopus* Oogenesis

**DOI:** 10.4172/2161-0436.S4-001

**Published:** 2012-05-05

**Authors:** Gregory A. Imbrie, Hao Wu, David C. Seldin, Isabel Dominguez

**Affiliations:** Hematology-Oncology Section, Department of Medicine, Boston University Medical School, 650 Albany Street, Boston, MA, USA

**Keywords:** CK2α, Animal, *Xenopus* oocyte, RNA localization, Protein localization, 3’UTR, Coding, Asymmetry

## Abstract

The establishment of the dorso-ventral axis is a fundamental process that occurs after fertilization. Dorsal axis specification in frogs starts immediately after fertilization, and depends upon activation of Wnt/β-catenin signaling. The protein kinase CK2α can modulate Wnt/β-catenin signaling and is necessary for dorsal axis specification in *Xenopus laevis*. Our previous experiments show that CK2α transcripts and protein are animally localized in embryos, overlapping the region where Wnt/β-catenin signaling is activated. Here we determined whether the animal localization of CK2α in the embryo is preceded by its localization in the oocyte. We found that CK2α transcripts were detected from stage I, their levels increased during oogenesis, and were animally localized as early as stage III. CK2α transcripts were translated during oogenesis and CK2α protein was localized to the animal hemisphere of stage VI oocytes. We cloned the CK2α 3’UTR and showed that the 2.8 kb CK2α transcript containing the 3’UTR was enriched during oogenesis. By injecting ectopic mRNAs, we demonstrated that both the coding and 3’UTR regions were necessary for proper CK2α transcript localization. This is the first report showing the involvement of coding and 3’UTR regions in animal transcript localization. Our findings demonstrate the pre-localization of CK2α transcript and thus, CK2α protein, in the oocyte. This may help restrict CK2α expression in preparation for dorsal axis specification.

## Introduction

The establishment of the embryonic axes is a fundamental process that dictates the subsequent development of the body plan. In a number of species, the dorsoventral axis is set before the first cleavage through the repositioning of molecules that are asymmetrically distributed during oogenesis [1,2]. In *Xenopus laevis*, the molecules essential for the development of dorsal structures are called dorsal determinants. The dorsal determinants are found vegetally in oocytes [3-5], and their composition is beginning to be understood [6-8]. Shortly after fertilization, the rotation of the egg cortex relative to the underlying cytoplasm moves the dorsal determinants to the prospective dorsal side of the embryo [5]. This cortical rotation is essential for embryonic development because if inhibited, the embryo does not develop any differentiated structures or embryonic axes [5]. At the end of cortical rotation, the dorsal determinants lie in contact with the equatorial cytoplasm. It is thought that the interaction between the dorsal determinants and the equatorial (marginal) cytoplasm activates the dorsal program [9]. This interaction results in the activation of Wnt/β-catenin signaling [10,11], a pathway required for dorsal axis formation in *Xenopus laevis* [12-14].

A number of components of Wnt/β-catenin signaling have been identified to play a role in dorsal axis formation in *Xenopus laevis* [12]. We recently identified CK2 as a bona fide component of canonical Wnt signaling that is necessary and sufficient for *Xenopus laevis* dorsal axis formation [15]. CK2 is required for the stabilization of the key component of Wnt/β-catenin signaling, β-catenin [15-18]. Intriguingly, transcript and protein for the catalytic (α) and regulatory (β) subunits of CK2 are enriched in animal blastomeres of the *Xenopus* morula [19] and are present in animal and equatorial blastomeres in the *Xenopus* blastula [15,19]. This precise CK2 localization may be required for proper regulation of β-catenin, as the distribution of endogenous CK2 [15] overlaps the equatorial region where β-catenin upregulation occurs in the embryo [11,20].

Asymmetric transcript and protein localization along the animal-vegetal axis of the *Xenopus* embryo is typically maternally derived from the oocyte [21-25]. During *Xenopus laevis* oogenesis, a number of transcripts are found localized to the vegetal or animal hemispheres. Vegetally enriched transcripts code for proteins that are essential for dorsal axis formation, e.g. dorsal determinants such as *XWnt11* [26], germ layer specification, e.g. *VegT* [27-29], germ cell lineage specification, e.g. *Xdazl* [30] and cell movements, e.g. Vg1 [31,32]. Two mechanisms have been proposed for vegetal localization of transcripts during oogenesis, an early and a late pathway (reviewed in [33-35]). The early pathway starts at stage I and is responsible for the localization of transcripts involved in germ cell determination. The late pathway starts around stage II and is responsible for the localization of dorsal determinants and mesendodermal determinants. Both pathways require transcript-specific sequences in the 3’ untranslated regions (UTR) for proper transcript localization [33-35].

Animally located transcripts in *Xenopus laevis* are important for embryonic germ layer specification, e.g. *POU60/Oct- 60* [36,37], cell signaling, e.g. *XWnt5a*, [38], and cell polarity, e.g. par-1, [39]. A number of studies suggest a complex regulation of animal localization during *Xenopus laevis* oogenesis, however no mechanism for animal transcript localization has been described. Animally enriched transcripts may localize gradually throughout oogenesis, e.g. *Xbub3* [40] and *Xoom* [41] or late in oogenesis, e.g. *XGβ1* [42]. Furthermore, animally enriched transcripts are found localized to the animal subcortical layer, e.g. *Ets-1, Ets-2* [43], *XGβ1, PAPBP* [44], to the perinuclear region, e.g. *Xbub3, PABP* [44], or throughout the animal hemisphere, e.g. An3 [31]. In addition, in some cases both protein and transcripts are animally restricted, e.g. *Xoom* [ 41,45], in other cases, the protein is animally restricted while the transcripts are ubiquitous, e.g. IP3R [46]. In this study, our goal was to ascertain the dynamics of localization of the catalytic subunit of CK2, CK2α, during *Xenopus laevis* oogenesis.

## Materials and Methods

### Oocyte collection, culture, treatment and injection

Frogs were housed and used in accordance with relevant guidelines and regulations and according to a protocol approved by the Boston University Institutional Animal Care and Use Committee.

For oocyte isolation, ovaries surgically obtained from tricaine-anesthetized females were subjected to digestion with Liberase Blendzyme (Roche, 1.4 mg/ 10 ml) in OR2 (1X OR2 is 80 μM NaCl, 2.5 μM KCl_2_, 1 μM Na_2_HPO_4_, 3.8 μM NaOH, 5 μM HEPES, pH 7.8, sterile filtered) for 40 minutes. The oocytes were then washed 10 times with 50 ml 1X Modified Barth’s Saline (MBS) without Ca^2+^ and placed in Petri dishes (50-60 oocytes /per 60 mm dish) in 1X MBS (1X MBS is 88 μM NaCl, 1 μM KCl, 10 μM HEPES, 0.8 μM Mg_2_SO_4_, 0.3 μM Ca(NO_3_)_2_, 0.4 μM CaCl_2_, pH 7.6 sterile filtered). Where indicated in the text, stage V and VI oocytes were obtained by manual defolliculation. Oocytes were left to rest overnight before manipulation and were stored and maintained during experiments at 18°C in 1X MBS and the media was changed twice a day.

Oocytes were sorted into stages I to VI according to Dumont [47] based on features including pigmentation and diameter, and were visualized and photographed with a Leica MZ6 dissecting microscope. Oocytes were injected in 1X MBS with 5-10 nl of indicated solutions. The site of injection varied depending upon the experiment as indicated in the results section. To obtain mature oocytes, stage VI oocytes were treated *in vitro* with 1μM progesterone for 5-7 hours until GVBD (Germinal Vesicle Breakdown) was morphologically visible as a white spot in the animal pole of the oocyte. GVBD was confirmed by the absence of nuclei after TCA fixation [48].

### Whole mount *in situ* hybridization (WISH)

De-folliculated pigmented oocytes were fixed in fresh MEMFA (MOPS 0.1 M pH 7.4, EGTA 2 mM, 1 mM MgSO4, 1.4% formaldehyde) for 1 hour. After fixation, oocytes were dehydrated with MeOH in graded steps to 100% and stored at -20°C. *In situ* hybridization was performed as described in [15]. Hybridized probe appears as purple staining. For photography, oocytes were partially bleached and cleared.

### Oocyte bisection for RNA and protein analysis

Before bisection for RNA isolation, oocytes were lightly fixed to avoid loss of cytoplasmic content. Oocytes at stages III and IV were incubated for 10 minutes in 1X MBS/10% methanol. Oocytes at stages V, VI, and matured oocytes were incubated for 5 minutes in 1X MBS/10% methanol. Ooocytes were stripped of their vitelline envelope with forceps. In the case of the oocytes, this eliminated the potential contamination of oocytes with any remaining follicle cells. Oocytes were then laid on their side and cut into animal and vegetal halves with a steel knife. During cutting, the oocyte halves seal shut and the cytoplasmic contents are preserved. Halves were snap frozen on dry-ice and stored at -80°C.

For protein analysis, oocytes were fixed in MEMFA for 18 minutes based on the study of [11]. After fixation, oocytes were washed three times in 1X MBS. Oocytes were stripped of their vitelline envelope and cut into animal and vegetal halves and frozen as described above. During cutting, the halves remain open however there is no cytoplasmic loss. Halves were snap frozen on dry-ice and stored at -80°C.

### RNA isolation and analysis of transcript copy number

For RNA isolation, pools of five (oocytes at stages V or VI) or ten (oocytes at stage III and IV) halves were processed. RNA was extracted with Trizol^®^ (Invitrogen), DNAse I treated, and cDNA was prepared from 1 μg total RNA using the BioRad iScript cDNA Synthesis Kit according to manufacturer’s instructions. Priming was via random and oligo-dT priming. Control “-RT” samples were made with no reverse transcriptase in the reaction. Quantitative PCR (qPCR) was carried out in a 25 μl iTaq Sybr Green reaction (BioRad), in the presence of 400 nM of each primer in a Stratagene mx3000P real-time PCR machine. Samples were analyzed in duplicate. It was determined for each sample and copy number was determined using a standard formula: 10^(Ct-40)/-3.32)^. Transcript copy number was normalized to the copy number for ornithine decarboxylase (Odc).

Sequences of primers for qPCR were chosen using the Primer Express primer analysis program and each primer set was verified by performing qPCR amplification with the primers on cellular cDNA and plasmid DNA, if applicable. Primers with distinct dissociation curve peaks and linear quantitation of cDNA (over 10 two-fold dilutions) and plasmid DNA (over 10 two-fold dilutions) were used for experimental qPCR analysis. When possible, primers were chosen that spanned an intron. The following sequences were used for qPCR analysis. CK2α: (forward: 5’ AAAGATCCTGGAGAACCTGCG 3’; reverse: 5’ TGTTCGAAGACAAGTGCTGGC 3’); 3’UTR of CK2α: (forward: 5’ ATGAGCCTGATGCCCCATATC 3’; reverse: 5’ ACACATTCCATCAGTGCACCC 3’); *XWnt5a*: (forward: 5’ GGTTTGCCAAGGAGTTTGTCG 3’; reverse: 5’ TCCGGCCTCATTATTGTGGAG 3’); *XWnt11*: (forward: 5’ AGGACAGGCTGTGCAACAAGA 3’; reverse: 5’ TCCACGATGGTTTCGGTGTAG 3’); and GFP: (forward: 5’ TAAACGGCCACAAGTTCAGCG 3’; reverse: 5’ CGGTGGTGCAGATGAACTTCA 3’).

### Protein isolation and immunoblot analysis

For protein analysis, pools of 10 halves of stage VI oocytes were homogenized in 1X Laemmli buffer. Proteins were electrophoresed and electroblotted onto polyvinylidene difluoride (PVDF) membranes (Millipore) and quantitative immunoblotting analysis using Fluor-S MultiImager (BioRad) was performed as described in [15]. The volume equivalent of 0.2 stage VI oocytes (stage VI volume =1μl) was loaded for immunoblotting, as it was within the linear range of detection for all proteins studied (I. Dominguez, unpublished data).

### *Xenopus* CK2α 3’UTR Cloning

From a discontinuous megablast (NCBI database) to the mouse 3’UTR (G.A. Imbrie, unpublished data), an EST clone containing CK2α was identified: IMAGE ID: 4971090 (Genbank Accession #: CF289385/CF289386). This clone was sequenced. By homology alignment, it was found to contain the full-length CK2α gene including a 960bp 3’UTR, extending from base 1395 to 2356. The 3’UTR contains a polyadenylation signal. This clone was termed pCMV-Sport6-CK2α-3’UTR. The 960bp 3’ UTR fragment was isolated by PCR with Elongase, using a forward primer that introduced an XbaI site (GCATTCTAGATAGGAGCCATCACAGTTGACC) and a reverse primer that introduced a NotI site (GCATGCGGCCGCGATAAATAAAACCATGTTTATTTACACC).

### Northern blot analysis

The *Xenopus* CK2α probe was generated by MluI and NdeI restriction digestion of the pCMV-Sport6-CK2α-3’UTR plasmid which contained the CK2α-UTR clone, producing a 2060bp fragment containing the CK2α coding sequence, and 684bp of the 3’UTR. The digestion also produced a 296bp fragment of the most 3’ region of the 3’UTR; these fragments were isolated by gel extraction. Probes were labeled with α^32^P dCTP using Klenow, purified using a ProbeQuant G-50 Micro Column (Amersham Biosciences) and the percent incorporation was measured in a scintillation counter. Northern blots were hybridized with radioactive labeled probe using Stratagene QuikHyb Solution (catalog #201220) following the manufacturer’s recommended protocol. The transcripts detected on the Northern membranes were quantified in a phosphoimager (Bio-Rad GS505 Exposure platform, Molecular Imaging Screen-BI and GS-525 Molecular Imager System reader and analyzed with Multi-Analyst software).

### Generation of eGFP constructs

To generate pCS2-EGFP and pCS2-EGFP-CK2α, the GFP coding sequence was amplified by PCR from plasmid pEGFP-C1 with a forward primer containing a ClaI site (GGA TTC ATC GAT ATG GTG AGC AAG GG) and a reverse primer containing StuI and EcoRI sites (AGGCCT GAA TTC CTT GTA CAG CTC GTC). The product was ligated into the pCS2-CK2α plasmid [15] or pCS2 alone to form pCS2-EGFP-CK2α and pCS2-EGFP, respectively.

To generate pCS2-EGFP-CK2-3’UTR and pCS2-EGFP-3’UTR, the 3’UTR was amplified by PCR and the plasmids pCS2-EGFP and pCS2-EGFP-CK2α were digested with XbaI and NotI. This digestion removed the SV40 polyadenylation signal present in the pCS2 vector. The 3’UTR fragment was ligated into the linearized pCS2-EGFP and pCS2-EGFP-CK2α plasmids with Quick Ligase to form pCS2-EGFP-CK2-3’UTR and pCS2-EGFP-3’UTR.

## Results

### CK2α transcript levels and dynamic localization during oogenesis

**Figure 1:**
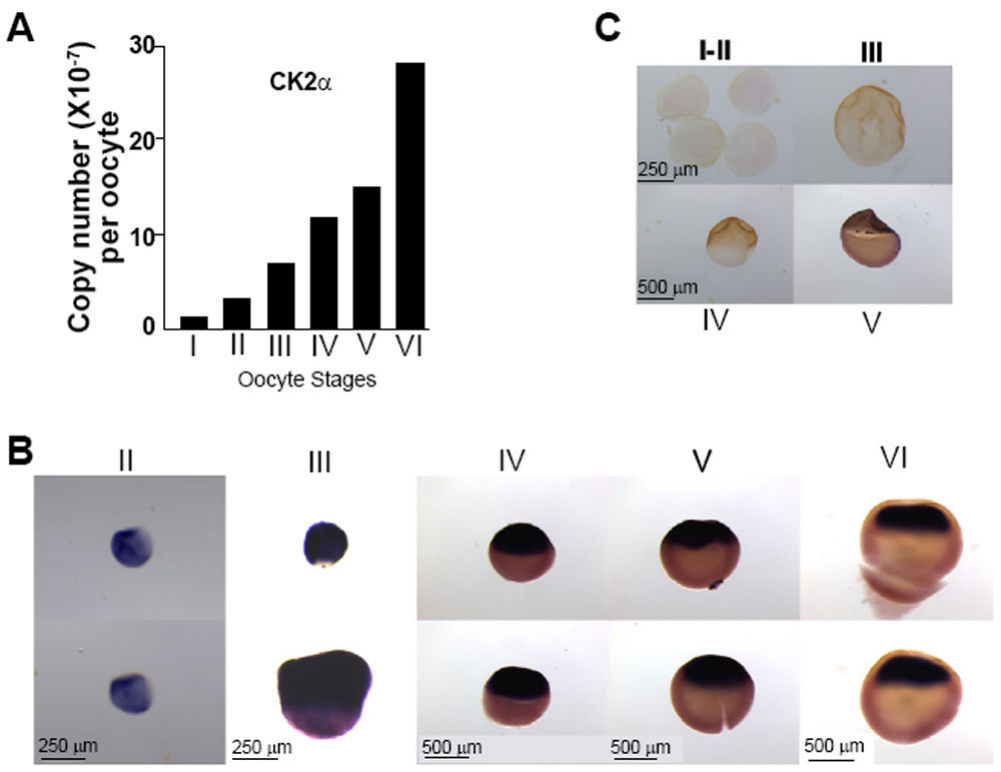
CK2α transcripts levels and localization by WISH (A) Analysis of total CK2α transcripts in *Xenopus* oocytes by RT-qPCR. Oocytes were collected, RNA extracted and quantitative PCR was carried out with specific primers to the CK2α coding sequence. The transcript for CK2α increases during oogenesis. This experiment was repeated twice with similar results. (B,C) Whole mount *in situ* hybridization of oocytes at different stages with an antisense CK2α -digoxigenin-labeled probe. (B) Chromogenic staining, shown in purple, indicates transcript localization. (C) Representative no probe controls for the *in situ* hybridizations. Animal hemisphere is up in all panels. Scale bars 250μm or 500μm as indicated in picture. This experiment was repeated using oocytes from three different frogs with similar results.

CK2α transcripts are enriched in the animal hemisphere of stage 3 embryos by whole mount *in situ* hybridization (WISH) [15] and realtime PCR (RT-qPCR, not shown). Since molecules with asymmetrical distribution in the early embryo typically inherit their patterns from the oocyte, we examined the levels and localization of CK2α transcripts during oogenesis. RT-qPCR analysis showed CK2α transcripts present and increasing in levels during oogenesis (Figure 1A). Next, we determined whether and when CK2α transcripts are animally distributed in the *Xenopus laevis* oocyte by WISH. As with RT-qPCR analysis, CK2α transcripts were detected throughout oogenesis. Enriched animal hemisphere staining was first observed in stage III oocytes, and clear animal hemisphere staining was found in stage IV and V oocytes (Figure 1B). In stage VI, staining was further restricted by exclusion from the animal cortex and subcortical cytoplasm. Stage VI oocytes matured *in vitro* showed similar staining to stage VI oocytes, like other animally localized transcripts that remain animally localized following oocyte maturation [22] (not shown). No-probe controls did not show staining (Figure 1C). These findings suggest that animal restriction of CK2α transcripts occurs in early oogenesis.

**Figure 2:**
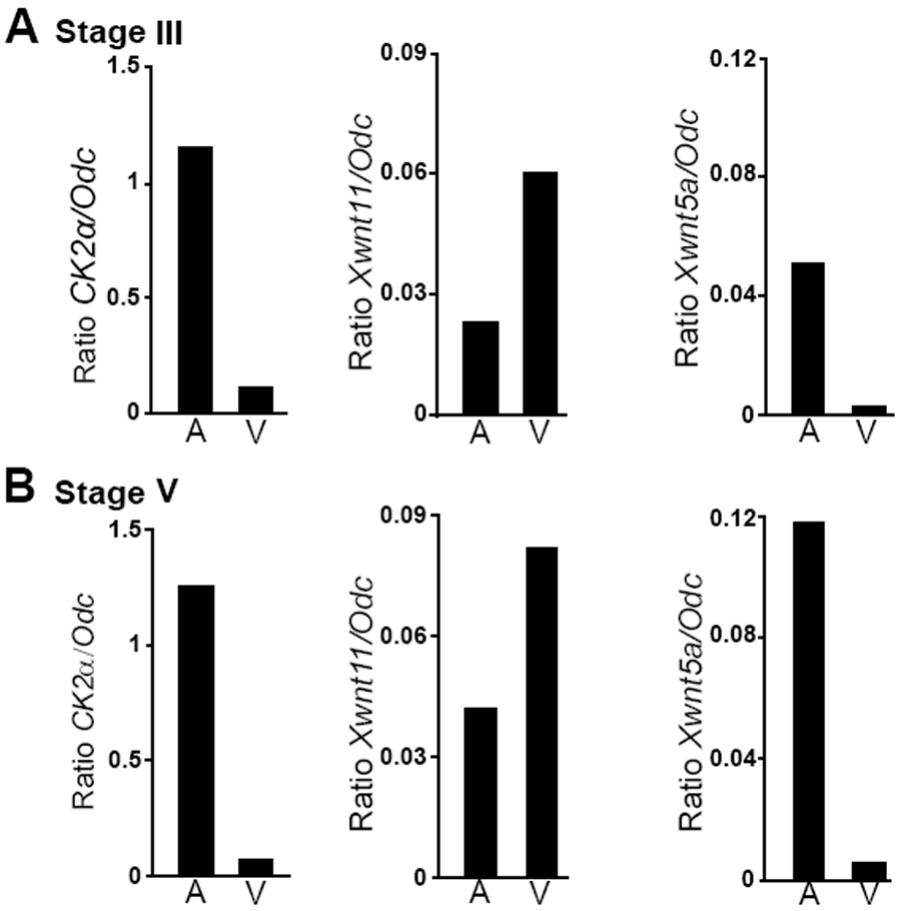
CK2α transcript localization by RT-qPCR RT-qPCR analysis of 10 pooled animal (A) or vegetal (V) halves at late stage III (A) and stage V (B) stages of oogenesis with specific primers for CK2α; *XWnt11*, a known vegetally localized transcript; *XWnt5*, an animally enriched transcript. From left to right, histograms represent transcript number normalized by *Odc* for CK2α, *XWnt11* and *XWnt5a*. This experiment was repeated three times for CK2α and *Odc*, and twice for Wnts with similar results.

As WISH sometimes fails to reveal vegetally located transcripts due to poor probe penetration through the yolk, we analyzed the localization of CK2α transcripts by RT-qPCR. Late stage III and stage V oocytes were divided into animal and vegetal portions as shown in figure S1A. As controls we examined *XWnt11* and *XWnt5a* transcripts (Figure 2A,B). *Odc* was used as a control as we found more evenly distributed throughout the oocyte than *Gapdh* [22]. Consistent with reported results, *XWnt5a* localized to the animal portion of the oocyte [38]. *XWnt11* transcripts were present both animally and vegetally, with enrichment in the vegetal hemisphere at stage III, in agreement with *in situ* hybridization analysis [26]. At stages V and VI, even though *in situ* hybridization shows exclusive vegetal localization [26], by RT-qPCR, *XWnt11* transcripts can also be found in the animal hemisphere and equatorially [22,49]. RT-qPCR analysis demonstrated that CK2α transcripts were preferentially localized animally at late stage III (Figure 2A), stage V (Figure 2B), stage VI and matured oocytes (not shown). Thus, CK2α transcripts localize to the animal hemisphere during oogenesis as early as stage III.

### CK2α protein is localized animally in oocytes

**Figure 3:**
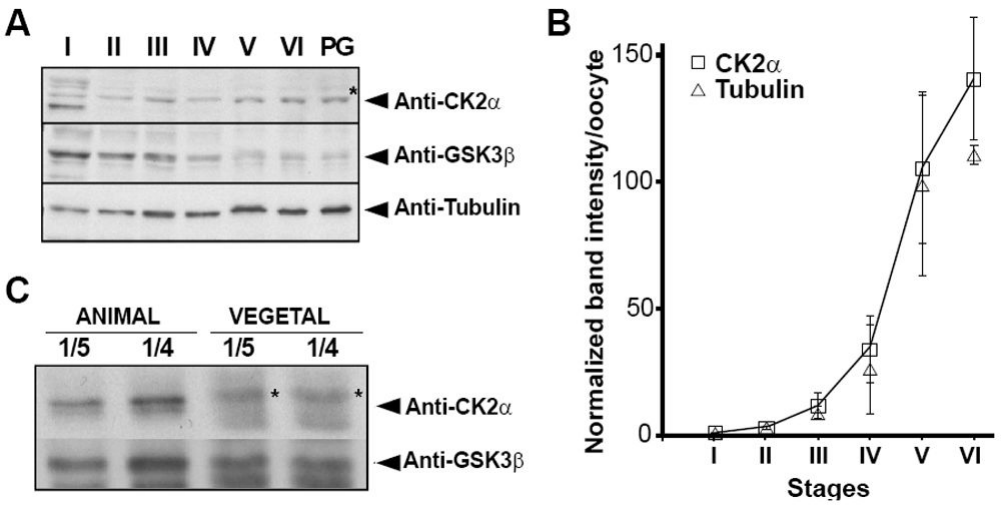
CK2α protein is enriched in the animal hemisphere of stage VI *Xenopus* oocytes (A) Expression of CK2 proteins during *Xenopus* oogenesis. Extracts were prepared from oocytes stages I, II, III, IV, V, VI, and stage VI treated with progesterone (PG). The volume equivalent of 0.2 stage VI oocytes was subjected to immunoblotting. Thus, we loaded, 22 stage I oocytes, 6.8 stage II oocytes, 2.5 stage III oocytes, 0.7 stage IV oocytes, 0.3 stage V oocytes and 0.2 stage VI oocytes. All immunoblots were repeated three times. The asterisk (*) indicates previously identified non-specific cross-reactive proteins in *Xenopus* extracts [15]. (B) Graph representing the data (band intensity) from the three independent experiments as in Figure 3A. Protein band intensity was divided by the number of oocytes loaded to obtain the band intensitity per oocyte, normalized to stage I oocyte (stage I =1), and represented as mean ±S.D. (C) Immunoblot analysis of CK2 protein levels in 10 pooled stage VI animal or vegetal halves. One fifth (1/5, 0.2 μl) and one fourth (1/4, 0.25 μl) of oocyte lysate were loaded for immunoblot analysis. The asterisk (*) indicates a non-specific cross-reactive protein in *Xenopus* extracts [15]. CK2α protein is animally enriched at stage VI of oogenesis, while other tested proteins are not. This is a representative experiment out of three with identical results.

CK2α protein was present at all stages of oogenesis and increased overtime indicating that the transcript is continuously translated (Figure 3A). In contrast, the transcript for another serine threonine kinase GSK3β decreased during oogenesis compared to tubulin (Figure 3A). Using these data, we calculated the protein content per oocyte. CK2α and tubulin increase approximately 125 fold from stage I to stage VI, what is proportional to the increase in oocyte volume (Figure 3B).

During *Xenopus* oogenesis asymmetrically distributed proteins can be found along the animal-vegetal axis of the oocyte [23,24,50,51]. We examined the animal/vegetal distribution of CK2α protein in stage VI oocytes by immunoblot of animal and vegetal halves obtained by cutting oocytes along the pigmentation line (Figure S1B). Immunoblot analysis showed that CK2α was localized in the animal half of the stage VI oocyte while XGSK3β was detected in both animal and vegetal halves (Figure 3C). Thus, CK2α protein localizes to the animal hemisphere of full-grown stage VI oocytes.

### CK2α transcripts in oocytes and cloning of CK2α 3’UTR

**Figure 4:**
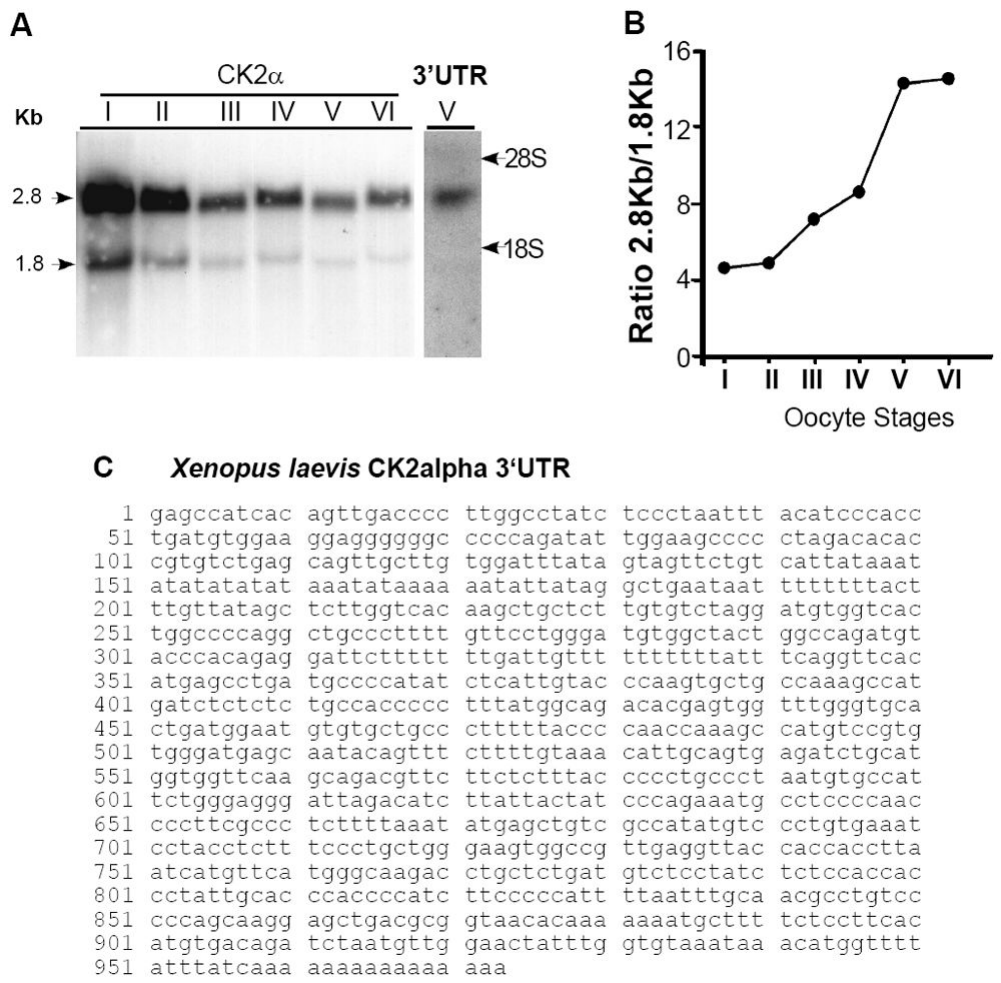
Analysis of CK2α transcripts during oogenesis and sequence of the XCK2α 3’UTR (A) Northern blot analysis of RNA extracted from different stages of *Xenopus laevis* oogenesis using a CK2α coding sequence probe (CK2α, left columns) or probe specific to the CK2α 3’UTR (3’UTR, right column). Analysis shows two major CK2α transcripts, estimated at 2.8 kb and 1.8 kb. The upper band was identified as containing the 3’UTR sequence. Position of the 28S and 18S rRNA bands is marked. The northern blots were performed twice with similar results. As younger oocytes express less RNA than late oocytes, we loaded RNA from 2.5 stage I oocytes, 1.3 stage II oocytes, 0.52 stage oocytes III, 0.08 stage IV oocytes, 0.03 stage V oocytes and 0.01 stage VI oocytes. (B) Graph representing the ratio of the 2.8kb to 1.8kb XCK2α transcripts quantified from the Northern blot in Figure 4B. (C) An EST clone containing a XCK2α and its 3’UTR was identified (IMAGE ID: 4971090, BC072167 / Genbank Accession #: CF289385/CF289386) and sequenced. The 3’UTR was defined as starting at the first base downstream of the stop codon.

We analyzed CK2α transcript size during oogenesis by Northern blot using a CK2α probe. Two major CK2α transcripts of approximately 2.8 kb and 1.8 kb were detected (Figure 4A), similarly to those reported in adult tissues [52]. The ratio of 2.8 kb and 1.8 kb CK2α transcripts increased 3-4 fold from stage I to stage VI oocytes (Figure 4B).

The 2.8 kb transcript was identified as containing the 3’UTR by probing a Northern blot with a 296 pb fragment of the CK2α 3’UTR (X3’UTR) (Figure 4A). For this, we identified the CK2α EST clone containing the 3’UTR from a discontinuous megablast (NCBI database) to the mouse 3’UTR (Figure 4C). We obtained the *Xenopus* clone (CF289385/CF289386) from the IMAGE consortium and used it to isolate the 960 bp 3’UTR sequence used as a northern probe (X3’UTR). Since the 2.8 kb transcript is favored during oogenesis, this supports the idea that the 3’UTR may be playing a role in CK2α function during oogenesis.

### Coding and 3’UTR regions are necessary for localization

**Figure 5:**
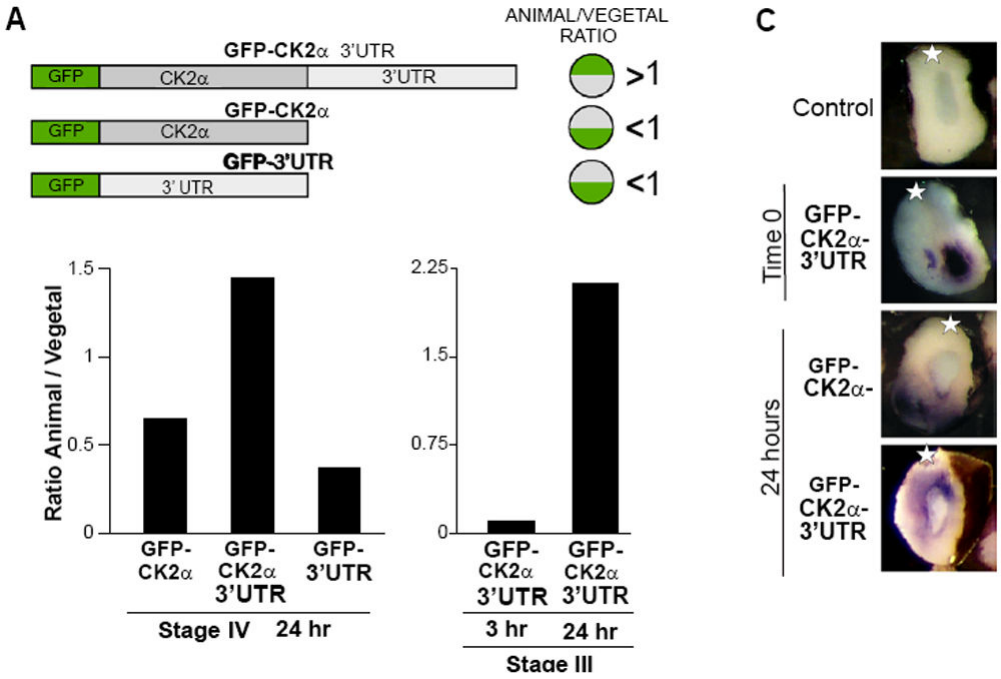
CK2α coding and 3’UTR transcript regions are necessary for ectopic mRNA localization (A) On the left, schematic representation of the constructs GFP-CK2α-3’UTR, GFP-CK2α and GFP-3’UTR. On the right, summary of the results presented in this figure. (B) Histograms depicting the animal to vegetal ratio of ectopic mRNAs. Oocytes were injected vegetally with 1 ng of *in vitro* transcribed mRNA from the constructs GFP-CK2α-3’UTR, GFP-CK2α and GFP-3’UTR and five animal and vegetal halves were processed for RT-qPCR to the GFP sequence. The animal to vegetal ratio of GFP copy number was calculated (Left histogram) 48 hrs after injection of mRNAs from all the constructs into stage IV, and (Right histogram) 3 and 24 hrs after injection of GFP-CK2α-3’UTR mRNA into stage III. The CK2α-3’UTR mRNA relocalized animally while CK2α and 3’UTR mRNAs did not relocalize. These experiments were repeated twice with similar results. (C) Localization of digoxin-labeled ectopic mRNAs twenty-four hours after vegetal injection. Stage IV oocytes were injected vegetally with 1 ng of *in vitro* transcribed digoxigenin-labeled sense mRNA from the GFP-CK2α-3’UTR and GFP- CK2α constructs (time 0). 24 hours after injection, oocytes were fixed, stained with anti-digoxigenin antibodies as whole-mounts, and bisected with a steel knife for photomicrography. Chromogenic staining is shown in purple. Uninjected oocytes showed no staining (control). * = animal. The CK2α-3’UTR mRNA relocalized animally while CK2α mRNA did not relocalize. This experiment was repeated twice with similar results.

In *Xenopus*, injected ectopic mRNAs containing the 3’UTR localize in a similar pattern to their endogenous counterparts [53,54]. We used the same strategy to determine whether the 3’UTR of CK2α plays a role in animal localization of CK2α transcripts. For this, three GFP-expressing plasmid constructs were engineered (Figure 5A): one containing only the CK2α 3’UTR (GFP-3’UTR); one containing the CK2α coding region alone (GFP-CK2α); and one containing both (GFP-CK2α-3’UTR). The SV40 polyadenylation sequence present in the original plasmid was removed during the generation of the GFP-3’UTR and GFP-CK2α-3’UTR plasmids. mRNAs were transcribed *in vitro* from the three plasmids and injected into the vegetal hemisphere of stage IV oocytes. After 48 hours, the animal/vegetal (A/V) ratio of the mRNAs was calculated to assess ectopic transcript relocalization by RT-qPCR to the GFP sequence. mRNAs that relocalize to the animal hemisphere would have an A/V ratio of more than one, while mRNAs that do not relocalize would have an A/V ratio less than one. By 48 hours, the GFP-CK2α-3’UTR mRNA had an A/V ratio of 1.45, suggesting that it was relocalized to the animal hemisphere (Figure 5B, left panel). In contrast, the GFP-CK2α mRNA lacking the 3’UTR had an A/V ratio of 0.65, suggesting that it was not competent for relocalization (Figure 5B, left panel). The GFP-3’UTR mRNA had an A/V ratio of 0.4, also indicating that these mRNAs remained in the vegetal portion of the oocyte (Figure 5B, left panel). These results are summarized in Figure 5A (right panel). The GFP-CK2α-3’UTR mRNA also relocalized animally in stage III oocytes 24 hours after injection (Figure 5B, right panel). We corroborated the animal relocalization of GFP-CK2α-3’UTR mRNA by injecting labelled mRNAs. For this, digoxigenin-labelled mRNAs were injected vegetally into stage IV oocytes and cultured for 24 hours and whole-mount immunohistochemistry with and anti-digoxigenin antibody performed. mRNA transcribed from the GFP-XCK2α plasmid served as a control. Figure 5C shows that the GFP-CK2α-3’UTR mRNA relocalized to the animal hemisphere while the GFP-CK2α mRNA did not relocalize from its injected position in the vegetal hemisphere. These findings indicate that both the coding region and the 3’UTR are required for proper animal localization of CK2α transcripts.

## Discussion

Asymmetric localization of mRNA and proteins plays an important role in embryonic development; e.g. in the establishment of the body axes, specification of the germ layers and germ cell lineage, and the control of cellular processes such as cell fate determination, cell polarity and cell movement [13,55-63]. Our understanding of the mechanism of dorsal axis formation is limited because of an incomplete understanding of the nature, distribution and activation of the factors that regulate dorsal axis formation [12-14]. Dorsal axis formation in *Xenopus laevis* embryos depends upon asymmetric stabilization of β-catenin, the transcriptional co-activator of the canonical Wnt signaling pathway. Our previous data showed that CK2 can regulate β-catenin protein stability, and it is an essential regulator of dorsal axis formation in *Xenopus laevis* embryos [15-17]. CK2α/β is present in the right place, the equatorial region of the embryo, and at the right time to regulate β-catenin [15,19]. Since CK2α/β proteins and transcripts are animally localized in the embryo, the primary goal of this study was to determine whether CK2 protein and transcript localization is derived maternally. For this, we studied CK2α as it is the catalytically active protein subunit.

The experiments presented here show that animal hemisphere enrichment of CK2α transcripts occurs as early as stage III of oogenesis and it is maintained until stage VI. Animal localization in early oogenesis as has been also described for *Xbub3* [40] and *Xoom* [41] while others, such as XGβ1 [42], localize late in oogenesis. This suggests that, like vegetally localized transcripts, there may be early and late pathways for transcript localization. Our data shows that CK2α transcript localization during oogenesis can explain the transcript distribution in early *Xenopus laevis* embryos. Interestingly, at stage VI, CK2α transcript is excluded from the cortex and subcortical cytoplasm, potentially to ensure that CK2α distribution will not be altered due to cortical rotation after fertilization. However, during early embryogenesis, CK2α transcripts are also present in the most cortical part of the embryo, albeit in a lesser amount than in the inner cytoplasm [15], showing that redistribution of CK2α transcripts happens after cortical rotation.

CK2α protein was animally located suggesting that pretranslational localization of the transcript may establish the asymmetric distribution of CK2α protein. Of note, animal transcript localization does not preclude a contribution of post-translational localization of the proteins, as in the case of XaPKC, ASIP/PIR-3 [64]. CK2α protein levels increase during oogenesis corresponding to the reported increase in total CK2 activity during oogenesis [52]. The magnitude of change of CK2α protein and transcript levels during oogenesis differ, suggesting that CK2α may be regulated through translational or posttranslational mechanisms that could include phosphorylation by CK2α [65].

Additionally, we showed an enrichment of the 2.8 kb CK2α transcript containing the 3’UTR during oogenesis, correlating with increased animal localization of the transcript. A similar shift in CK2α transcripts ratio was observed in quiescent versus proliferating mouse cells (G. A. Imbrie, unpublished data) suggesting that the ratio between the transcripts may play a role in the regulation of CK2α in diverse systems, including other animal models.

Our data shows that CK2α transcript localization requires the coding and 3’UTR regions, which is a novel mechanism for RNA localization in *Xenopus* oocytes, as vegetal transcript localization depends exclusively on 3’UTR sequences [66]. Although this mechanism is novel for *Xenopus*, recent work identifies additional regions involved in RNA localization in other models. For example, the 5’UTR for *gurken* localization in *Drosophila* oocytes and *Glutelin* in *Oryza sativa*; and the coding region for *ASH1* localization at the budding tip in *Saccharomyces cerevisiae* and the *10-kDa δ-zein* RNA in *Oryza sativa* [67-69]. Since transcripts and/or proteins for other regulators of dorsal axis specification, such as *CK2β, XGSK3β, dishevelled*, Apc, axin, *Tcf-3* and *β-catenin* are, like CK2α, animally restricted [15,22,49], it will be interesting to test whether their localization also depends upon coding and 3’UTR sequences. Given the diversity in the timing and distribution patterns of animally localized transcripts and proteins, it is possible that diverse mechanisms may be used for their localization in the *Xenopus* oocyte. For example, *Ets-1, Ets-2* localize subcortically [43], *Xbub3* localizes perinuclearly [40], and some mRNAs and proteins localize dynamically during oogenesis between cellular regions, e.g. PABP [44]. Based on the importance of animally located transcripts in embryonic development, it will be important to determine the molecular and biochemical mechanism(s) of animal localization (e.g. the RNA sequences required for localization, the role of RNA-binding proteins, the dependence on active transport or anchoring to cellular structures and the contribution of the proposed transcript degradation in the vegetal hemisphere [70]).

**Figure 6:**
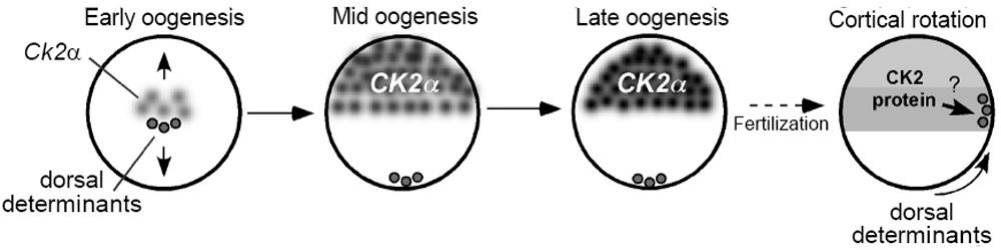
CK2α localization and dorsal axis formation. During oogenesis, CK2α mRNA is localized to the animal pole, in a process dependent on its coding and 3’UTR sequences, while the dorsal determinants are localized to the vegetal pole. Fertilized embryos will inherit the dorsal determinants and CK2 protein in non-overlapping regions that will come together after cortical rotation in the region where dorsal specification will be initiated.

In summary, we found that CK2α transcript and protein are localized to the animal hemisphere during *Xenopus laevis* oogenesis. These data are consistent with a model (Figure 6) in which maternal localization ensures that CK2α is localized animally while the dorsal determinants are located vegetally. Cortical rotation after fertilization relocalizes the dorsal determinants to the dorsal equatorial region of the embryo, overlapping the region where CK2 proteins are enriched. CK2 will act on the dorsal determinants to upregulate β-catenin and initiate dorsal axis formation. Although many questions remain to be answered, our results on CK2α demonstrates the importance for both the coding and 3’UTR sequences for localization, and therefore may serve as a model for the study of other animally distributed transcripts in *Xenopus laevis* and other model organisms.
